# Systematic Review of Sequencing Studies and Gene Expression Profiling in Familial Meniere Disease

**DOI:** 10.3390/genes11121414

**Published:** 2020-11-27

**Authors:** Alba Escalera-Balsera, Pablo Roman-Naranjo, Jose Antonio Lopez-Escamez

**Affiliations:** 1 Otology & Neurotology Group CTS 495, Department of Genomic Medicine, Centro Pfizer-Universidad de Granada-Junta de Andalucía de Genómica e Investigación Oncológica, 18016 Granada, Spain; alba.escalera@genyo.es (A.E.-B.); pablo.roman@genyo.es (P.R.-N.); 2 Department of Otolaryngology, Instituto de Investigación Biosanitaria, ibs.GRANADA, Hospital Universitario Virgen de las Nieves, 18014 Granada, Spain; 3 Department of Surgery, Division of Otolaryngology, Universidad de Granada, 18016 Granada, Spain

**Keywords:** Meniere’s disease, exome sequencing, sensorineural hearing loss, vestibular disorders, familial segregation, single nucleotide variant, rare variant, Mendelian disorders, inheritance pattern

## Abstract

Familial Meniere Disease (FMD) is a rare inner ear disorder characterized by episodic vertigo associated with sensorineural hearing loss, tinnitus and/or aural fullness. We conducted a systematic review to find sequencing studies segregating rare variants in FMD to obtain evidence to support candidate genes for MD. After evaluating the quality of the retrieved records, eight studies were selected to carry out a quantitative synthesis. These articles described 20 single nucleotide variants (SNVs) in 11 genes (*FAM136A*, *DTNA*, *PRKCB*, *COCH*, *DPT*, *SEMA3D*, *STRC*, *HMX2*, *TMEM55B*, *OTOG* and *LSAMP*), most of them in singular families—the exception being the *OTOG* gene. Furthermore, we analyzed the pathogenicity of each SNV and compared its allelic frequency with reference datasets to evaluate its role in the pathogenesis of FMD. By retrieving gene expression data in these genes from different databases, we could classify them according to their gene expression in neural or inner ear tissues. Finally, we evaluated the pattern of inheritance to conclude which genes show an autosomal dominant (AD) or autosomal recessive (AR) inheritance in FMD.

## 1. Introduction

The human inner ear is formed by six sensory organs: the spiral organ of Corti, located in the anterior part of the temporal bone, and the vestibular organs that consist of the utricle, the saccule, and the three semicircular canals that form the posterior labyrinth. These organs share a highly specialized tissue, the neurosensory epithelium, which contains the auditory and vestibular hair cells (HCs). The displacement of HC stereocilia opens the mechanotransduction channels at the hair bundle that mediates the conversion of mechanical signals to neural impulses at the afferent synapses to drive acoustic or acceleration information to the cochlear or vestibular nuclei in the brainstem [1,2].

Meniere Disease (MD) is an inner ear disorder that is characterized by episodic vertigo and associated with sensorineural hearing loss (SNHL), tinnitus and/or aural fullness. It is a multifactorial disorder where the combined effect of genetics and environmental factors probably determine the onset of the condition. The criteria to diagnose MD are based on the clinical symptoms occurring during the attacks of vertigo and the documentation of SNHL by a pure tone audiogram before, during or after the episode of vertigo [3]. Notably, MD and vestibular migraine, whose clinical features could overlap, are the most common causes of spontaneous recurrent vertigo [4].

Histopathological and MRI studies have demonstrated an enlargement of the endolymphatic space in patients with MD, with an accumulation of endolymph in the saccule and the cochlea—termed as endolymphatic hydrops [5].

The prevalence of MD in a population changes according to the geographical region and the ethnic background. Epidemiology records report a prevalence of MD that varies from 17 to 200 cases/100,000. In general, MD is more common in European descendants [6], and the age of onset ranges from 30 to 70 years. Moreover, MD is associated with several comorbid conditions such as autoimmune arthritis, psoriasis, irritable bowel syndrome and migraine [7].

Hierarchical clustering methods have identified five clinical subgroups of patients with MD, one of them being familial MD (FMD) [8,9]. FMD is a rare disease that is defined if at least another family member in the first or second degree, in addition to the proband, fulfills all the clinical criteria of definite or probable MD [3].

In populations of European descent, the FMD represents 8–9% of cases [10,11]. The pattern of inheritance is autosomal dominant (AD) in most families; however, genetic heterogeneity is found and recessive inheritance has also been reported [10]. A family history where several members have SNHL, migraine or recurrent vertigo, justifies the investigation of the proband and their relatives to verify if any of them accomplish the diagnostic criteria for MD [3].

Early studies in multiplex families with FMD using microsatellite markers and segregation analysis pointed to different loci [12,13,14], but no causal gene was identified. However, the development of exome sequencing has facilitated the identification of few novel candidate genes in singular families [15,16,17].

The aim of this systematic review is to critically analyze the evidence to support causal genes in FMD and to describe the potential role of these genes in the pathogenesis of MD by retrieving information from gene expression databases.

## 2. Materials and Methods

### 2.1. Study Design

This is a systematic review of sequencing studies in FMD, which follows Preferred Reporting Items for Systematic Reviews (PRISMA) guidelines (Supplementary Table S1) [18]. We also conducted an additional search in general and tissue specific databases to retrieve gene expression profiles of candidate genes for FMD. 

### 2.2. Research Question and Selection Criteria

This systematic review aims to describe genes reported in FMD in sequencing studies and to find which cell types and extracellular structures are potentially involved. Therefore, we formulated the question “which genes have been found to be associated with FMD?”. In concordance with the methodology for systematic reviews, this question was answered following the PICOS strategy:

- Population: Patients diagnosed with FMD.
- Intervention: Sequencing studies (Sanger or exome/genome sequencing) in FMD searching for rare variants to target candidate genes.
- Comparison: Allelic frequency (AF) was compared with population specific reference datasets (gnomAD: Genome Aggregation Database, CSVS: Collaborative Spanish Variant Server, SweGen and ExAC: Exome Aggregation Consortium) [19,20,21,22], for candidate variants in selected genes.
- Outcome: Genetic findings and pathogenicity scores reported (rare variants, candidate genes).
- Study design: Familial segregation studies.

### 2.3. Search Strategies

The literature search was carried out on 11th August 2020 using the PubMed database with the following keywords: (familial [Title/Abstract] OR family [Title/Abstract] OR gene [Title/Abstract] OR genes [Title/Abstract] OR inheritance [Title/Abstract] OR variation [Title/Abstract] OR mutation [Title/Abstract]) AND (Meniere Disease [Title/Abstract] OR Meniere’s Disease [Title/Abstract]). The search was filtered by the last 20 years (2000–2020) and written in English, by configuring an advanced search on PubMed.

Gene expression profiles for the candidate genes retrieved from the different studies were analyzed using the following datasets:

- Gene transcripts identified in the Neuropil and Somata layers of CA1 region in the Hippocampus in *Rattus norvegicus* by Cajigas et al. [23].
- Human synaptic genes in SynaptomeDB [24].
- Transcriptome catalogue of adult human inner ear, and the list of preferentially expressed mRNA genes in the inner ear when compared to 32 other tissues [25].

Additionally, the following datasets were obtained from the gEAR portal (https://umgear.org/):

- RNA-Seq in embryonic day 16.5 (E16.5) and postnatal day 0 (P0) from cochlear and vestibular sensory epithelium in mouse [26].
- RNA-Seq in P0 from cochlea and vestibule in mouse, where HCs were compared with epithelial non-HCs [27].
- RNA-Seq in P0 from cochlea in mouse to contrast HC with the rest of cochlear duct [28].
- RNA-Seq in adult mice from organ of Corti, comparing inner HCs (IHC), outer HC (OHCs), Deiters’ cells and pillar cells [29].
- Single cell RNA-Seq in postnatal day 1 (P1) and 7 (P7) from organ of Corti in mouse [30].

Furthermore, the SHIELD database [31] was used to retrieve the following datasets:

- RNA expression by microarray from adult mice from cochlea to analyze IHC and OHC [32].
- RNA expression by microarray from developmental stages of mice from spiral ganglion (SG) and vestibular ganglion (VG) [2].

### 2.4. Exclusion Criteria

Articles that accomplish the following characteristics were excluded from the systematic review:

- Animal studies were excluded from the first analysis.
- Studies not published in English.

### 2.5. Quality Assessment of Selected Studies

After screening titles and abstracts of the selected records, reviews, meta-analysis, and irrelevant records (non-genetic studies, pharmacogenomics or clinical studies) were removed. Articles that did not meet the eligibility criteria were discarded. The criteria were composed by three main questions:

- Is the study performed with two or more members of a family diagnosed with MD or with patients from different families but all of them diagnosed with FMD?
- Has the study reported a gene or a position in the genome statistically significant when it was compared to genome reference datasets?
- Has the study used an accurate methodology and is it described with enough details to validate its findings?

If all these questions were answered with “yes”, the record was selected for synthesis. Next, each reported variant was assessed and classified as benign/likely benign/unknown significance/likely pathogenic or pathogenic according to the American College of Medical Genetics and Genomics (ACMG) criteria [33,34] and a Combined Annotation Dependent Depletion (CADD) score [35,36].

### 2.6. Data Extraction and Synthesis

From each article selected, the following information was extracted to perform the synthesis: first author’s last name, publication year, study design, population, number of patients in the study, sex of the patients, diagnostic criteria used for MD, sequencing method and genetic findings, gene and genomic position of the variant type (single nucleotide or structural variant). Moreover, the Reference Single Nucleotide Polymorphism (rs), the AF in gnomAD, CSVS, SweGen and ExAC, the pathogenicity score according to the ACMG criteria, the CADD score and the inheritance pattern were obtained or calculated. In this systematic review, the list of retrieved candidate genes and the classification of these genes according to their inheritance pattern were the main outcomes. Furthermore, the gene expression profile in neural or inner ear tissues was used to define the cell types involved with each gene.

## 3. Results

### 3.1. Selection and Characteristics of FMD Studies

**Figure 1:**
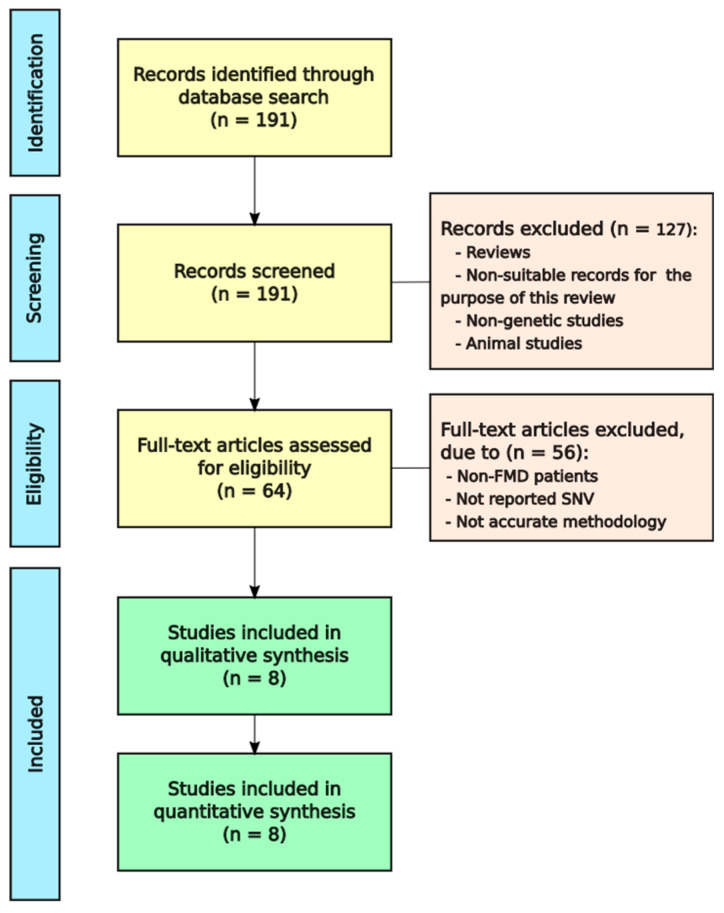
Flowchart to select Familial Meniere Disease (FMD) studies.

With the objective of knowing which of the rare variants or genes related to FMD, we obtained 191 articles from PubMed. Afterwards, reviews, meta-analysis and irrelevant records were removed. Finally, we evaluated if the 64 remaining articles met the eligibility criteria and eight of them were selected for qualitative synthesis (Figure 1).

One of the questions to accomplish the eligibility criteria was if all reported patients in each family were diagnosed as FMD, preferably they should be diagnosed following the diagnostic criteria described by the International Classification Committee for Vestibular Disorders of the Barany Society in 2015 [3]; however, only three of eight studies used these criteria; another three studies used the diagnostic criteria of the Committee on Hearing and Equilibrium of the American Academy of Otolaryngology–Head and Neck Surgery (AAO-HNS) published in 1995 [37], and two studies did not clarify the diagnostic criteria. One of them included three individuals in the same family with SNHL and episodic vertigo, and the other reported definite MD in two sisters without stating if they used the AAO-HNS or the Barany criteria to define MD. The diagnostic criteria of the Barany Society and the AAO-HNS are described in the Supplementary Table S2.

Among the eight selected studies, three articles reported candidate variants in two genes, one of them, *FAM136A* and *DTNA* in the same Spanish family, another record reported *DPT* and *SEMA3D* in two unrelated Spanish families and the other *HMX2* and *TMEM55B* in the same Finnish family. Five studies confirmed only one gene (*PRKCB*, *COCH*, *STRC*, *OTOG* and *LSAMP*) in families from Iran, South Korea, Spain and Sweden. 

All studies used Whole Exome Sequencing (WES), and seven of them also performed Sanger sequencing to validate the variants. Moreover, all of them have data about the population ancestry, the sex and the number of individuals affected. A summary of these studies is shown in Table 1.

### 3.2. Inheritance of SNVs Associated with FMD

Requena et al. [15] studied a family with three affected women in consecutive generations with definite MD. All cases segregated two heterozygous rare variants in *FAM136A* and *DTNA* genes, which suggests an AD pattern of inheritance. The variant chr2:70527974G>A in *FAM136A* (NM_032822.3) was a nonsense novel variant leading to a stop codon; the ultrarare heterozygous variant chr18:32462094G>T reported in the *DTNA* gene (NM_001390.4) produces an amino acid change from valine to phenylalanine and generates a novel splice-site sequence predicted as a constitutive acceptor. Both variants were classified as pathogenic (Table 2).

The novel missense variant in the *PRKCB* gene (NM_002738.7) [16] was found in a family with two cases of complete MD phenotype and the father of the proband with SNHL (incomplete phenotype), and the pattern of inheritance was considered AD with incomplete penetrance. The chr16:23999898G>T variant causes an amino acid change from glycine to valine and it was considered likely pathogenic.

The chr14:31349796G>A variant in the *COCH* gene was reported in a Korean family with DFNA9 phenotype [38]; of note, two siblings and her mother presented episodic vertigo and SNHL fulfilling criteria for definite MD and another two siblings had an incomplete phenotype. This SNV showed an AD inheritance pattern with incomplete phenotype and this family should be considered as FMD. This SNV is not described in gnomAD or ClinVar and it was classified as likely pathogenic. 

The SNV in the *SEMA3D* gene (NM_152754.2) [17] was found in a family with three individuals affected by MD in the same generation, all of them segregated the novel missense variant; in addition, there were another three individuals with incomplete phenotype in different generations. This variant, in chr7:84642128G>A, causes an amino acid change from proline to serine and it was classified as pathogenic. In the same article, Martín-Sierra et al. found a missense variant in the *DPT* gene (NM_001937.4) in a family with three sisters affected by MD, and one of the probands had anti-ribonucleoprotein antibodies, suggesting a comorbid immune disorder without all criteria for systemic lupus erythematosus.

Furthermore, in the family, seven relatives had incomplete phenotype (SNHL or episodic vertigo). The three patients with MD and two individuals presenting progressive bilateral SNHL and sudden SNHL, segregated the variant. The chr1:168665849G>A produces an amino acid change from arginine to cysteine and it was classified as likely pathogenic. Both SNVs had an AD inheritance pattern with an incomplete penetrance.

Frykholm et al. [39] described a family with two brothers and a first cousin with moderate, non-progressive SNHL and episodic vertigo. The two brothers shared a nonsense homozygous variant, which is chr15:43896948G>A, in the *STRC* gene (NM_153700.2), and the cousin had the same variant in heterozygosis inherited from the mother and a deletion of approximately 97 kb spanning the *STRC* gene, inherited from the father. None of the parents had symptoms of the disease, which suggests that it had an AR inheritance pattern.

Skarp et al. [40] found two heterozygous variants in *HMX2* and *TMEM55B*, which were present in an individual and his grandfather, both affected with MD. The father of the first individual did not report any symptoms of MD, and he would be an obligate carrier of these variants, but it was not possible to validate them because he did not donate a DNA sample for the study. Since these SNVs do not lead to MD with full penetrance, additional heterozygous variants in these genes should be considered to confirm recessive inheritance in this family. Both missense variants, chr10:124909634T>A in the *HMX2* gene (NM_005519.1) lead to an amino acid change from tyrosine to asparagine and chr14:20927370G>A in the *TMEM55B* gene (NM_001100814.2) from leucine to phenylalanine and were classified as likely pathogenic and with uncertain significance, respectively.

Roman-Naranjo et al. [41] reported several missense variants in the *OTOG* gene (NM_001277269.2) in multiplex Spanish families with FMD. Moreover, this study used a group of sporadic cases with MD, and AFs were compared with the non-Finnish European and Spanish (gnomAD) reference population datasets (CSVS). A heterozygous variant (chr11:17574758G>A), classified as pathogenic, was observed in two cases from two unrelated families; both families also shared the rare variant chr11:17663747G>A and one of them also had a third variant, chr11:17627548G>A. Moreover, two heterozygous variants, chr11:17578774G>A and chr11:17632921C>T, were found in four additional patients from four different unrelated families. In both sets of families, heterozygous compound recessive inheritance pattern was suggested. The rest of the variants were reported in three, two or one unrelated patients with FMD.

Recently, Mehrjoo et al. [42] studied a family with consanguineous parents with four descendants, two of them with definite MD and two unaffected siblings with an AR inheritance pattern. Both affected patients had poor senses of smell, which suggests that the phenotype could be MD-like phenotype. A novel homozygous variant chr3:115561402T>C in the LSAMP gene (NM_001318915) was reported in the two affected siblings with MD and it was classified as likely pathogenic. This SNV is not described in gnomAD or ClinVar.

### 3.3. Classification of Genes

**Figure 2:**
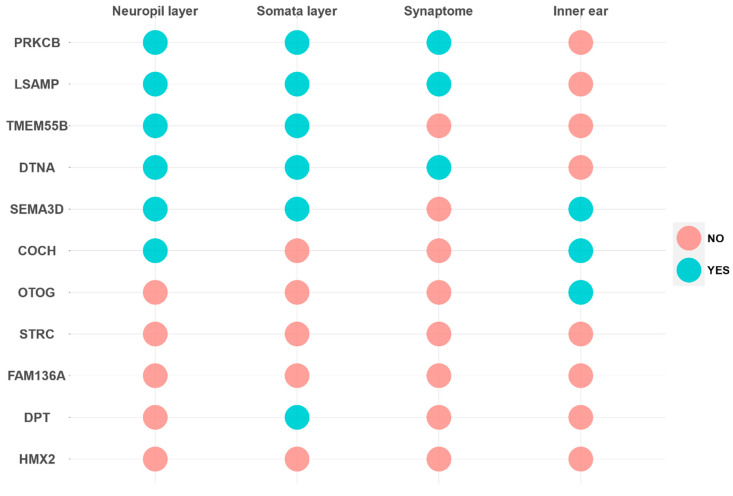
Summary of candidate FMD genes expressed in neural tissues and human inner ear databases, and in cyan genes that appear in the databases and in red genes that do not appear. Genes were identified in Neuropil layer and Somata layer of CAI Hippocampus in *R. norvegicus* (columns 1 and 2); genes that encode proteins of human synapsis (column 3) and preferentially expressed genes in the human inner ear compared to 32 other tissues (column 4).

**Figure 3:**
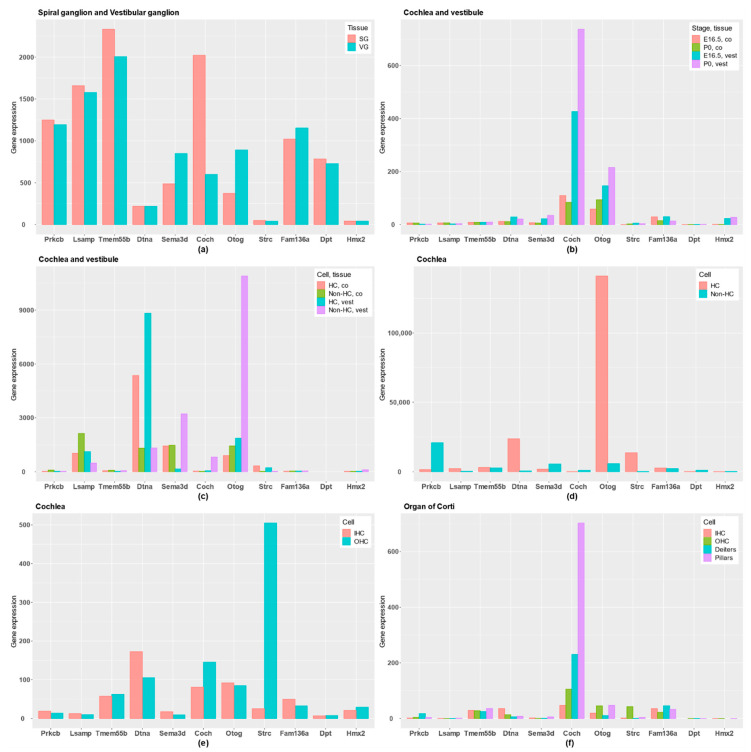
Gene expression profile in candidate genes for FMD in different tissues: (**a**) RNA expression data across inner ear developmental stages in mice comparing SG and VG; (**b**) RNA-Seq data comparing embryonic day 16.5 (E16.5) and postnatal day (P0) between cochlea (co) and vestibule (vest); (**c**) RNA-Seq data at postnatal day (P0) in mice comparing hair cells (HCs) with epithelial non-HCs in both cochlea (co) and vestibule (vest); (**d**) RNA-Seq data from postnatal day (P0) in mice comparing HCs with the rest of cochlear duct in cochlea; (**e**) RNA expression data from adult mice cochlea comparing inner HCs (IHCs) and outer HCs (OHCs); (**f**) RNA-Seq data from adult mice in the organ of Corti, comparing IHCs, OHCs, Deiters’ cells and pillar cells.

Genes were classified according to the gene expression profile in neural or the inner ear databases (Figure 2 and Figure 3).

First, *PRKCB* and *LSAMP* genes have gene expression in the three neural tissues and they are highly expressed in SG and VG, and they show a rather low expression in HCs. Remarkably, *PRKCB*, which encodes the β subunit of protein kinase C, shows a tonotopic distribution in tectal cells and inner border cells in the organ of Corti [16].

The *TMEM55B* gene is reported in neural tissues, the exception being the Synaptome database, and it is also highly expressed in both SG and VG. According to these data, the genes *PRKCB*, *LSAMP* and *TMEMB55B* will have a gene expression profile related to supporting cells in the neurosensory epithelia, sensory neurons or neural tissues.

However, *COCH* and *OTOG* encode the extracellular proteins cochlin and otogelin, and they are differentially expressed in the human inner ear. Moreover, gene expression datasets show that both are expressed in cochlea and vestibule; particularly, *OTOG* is expressed in non-HC of vestibular epithelia and cochlear HC in the organ of Corti, and *COCH* in the lateral wall of the cochlea, close to the organ of Corti. For these reasons, *COCH* and *OTOG* genes are predominantly expressed in inner ear tissues, although *COCH* was also reported in neuropil layer.

The *STRC* gene is not differentially expressed in the human inner ear, but it has a specific high expression in HCs, particularly in OHC, highlighting a key role in the OHC stereocilia in the organ of Corti.

*DTNA* presents a preferential gene expression in the three neural tissues and not in human inner ear, although the gene is expressed in both auditory and vestibular HCs in mouse, showing a low expression in non-HC. *SEMA3D* encodes the axonal guidance signaling protein semaphoring 3D, and it has an important role in neural tissues and in inner ear tissues. It is expressed in the human inner ear, but also in the Neuropil and Somata layers in neural tissues. In addition, it is higher expressed in non-HC than in HC, and is expressed in both SG and VG.

Finally, no conclusive gene expression data were found for *FAM136A*, *DPT* and *HMX2* genes, which have a low expression in inner ear tissues or neural tissues. However, the *DPT* gene seems to have a higher expression in the Somata layer.

**Figure 4:**
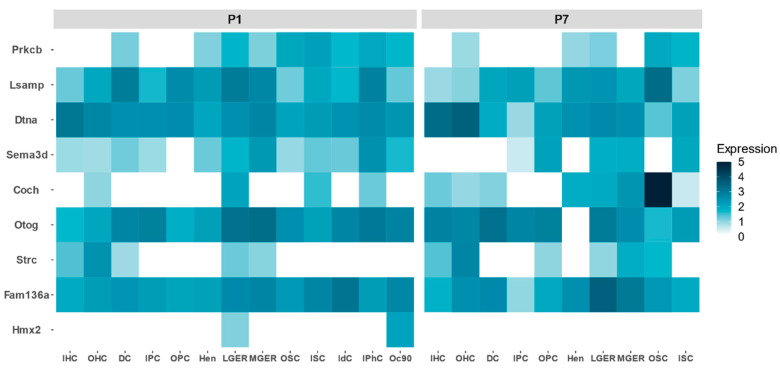
Relative gene expression of candidate genes for FMD in the mouse organ of Corti in postnatal days 1 (P1) and 7 (P7) comparing different cell types: inner hair cells (IHC), outer hair cells (OHC), Deiters’ cells (DC), inner pillar cells (IPC), outer pillar cells (OPC), Hensen cells (Hen), lateral greater epithelial ridge cells (LGER), medial greater epithelial ridge cells (MGER), outer sulcus cells (OSC), inner sulcus cells (ISC), interdental cells (IdC), inner phalangeal cells (IPhC), cells expressing Oc90 (Oc90).

FMD genes show a different expression profile during development in the mouse organ of Corti. By retrieving single cell gene expression data from P1 and P7, we could establish which cell types reveal a relatively high expression from early stages to adult inner ear (Figure 4). *COCH* is highly expressed in the organ of Corti, particularly in the outer sulcus cells. There are genes that show a high expression in P1, but their expression decreases at P7, such as *PRKCB*, *SEMA3D* or *LSAMP*, suggesting a relevant role during the maturation of the organ of Corti.

## 4. Discussion

### 4.1. Main Findings in FMD Candidate Genes

FMD is not a monogenic disorder. In this systematic review, we have found 11 candidate genes related with FMD, which supports the genetic heterogeneity of the condition. Although further evidence from cellular and animal models is needed, the finding of rare missense variants in the *OTOG* gene in multiplex unrelated families strongly support their role in the pathogenesis of FMD. Some SNVs in *OTOG* (chr11:17574758G>A, chr11:17578774G>A, chr11:17621218C>T, chr11:17632921C>T, chr11:17663747G>A and chr11:17667139G>C) [41] were reported in multiplex families with different unrelated individuals with FMD. Whereas SNVs in *PRKCB* [16], *DPT*, *SEMA3D* [17], *COCH* [38], *STRC* [39], *HMX2*, *TMEM55B* [40] and *LSAMP* [42] were only described in simplex families. Hence, it would be necessary to find new additional cases or families with these variations to support their involvement in FMD.

The heterogeneity observed in the genetics of FMD suggests that different causes could lead to the same syndrome, consisting of episodic vertigo associated SNHL and tinnitus during the attacks. This complex phenotype is the result of genetic and environmental factors and several genes will play a role in specific cell types in the inner ear, and other genes will encode essential neural signals to support the innervation of the sensorineural epithelia, such as *SEMA3D*. Therefore, one of the objectives of this systematic review is to classify the genes related to FMD in these two groups, according to their predominant gene expression profile.

### 4.2. Inheritance Pattern in FMD

In this systematic review, we have found evidence to support that the inheritance pattern in FMD could be AD or AR. As it was previously reported, some of these AD families have a partial syndrome (SNHL or vertigo). The SNVs in *FAM136A*, *DTNA*, *PRKCB*, *COCH*, *DPT* and *SEMA3D* have and AD inheritance pattern; specifically, there are variants in *PRKCB*, *COCH*, *DPT* and *SEMA3D* segregates with an incomplete penetrance because in the families reported, there were some relatives that presented an incomplete phenotype of MD.

On the other hand, SNVs in *STRC* and *LSAMP* had an AR inheritance pattern. Moreover, SNVs in *HMX2* and *TMEM55B* and five SNVs of *OTOG* also have an AR inheritance.

### 4.3. Classification of Genes

Familial MD genes with a predominant gene expression profile in neural tissues are *PRKCB*, *TMEM55* and *LSAMP*. It is possible that rare missense SNVs in these genes could contribute to the MD phenotype by affecting protein function and thus hindering neural development, maintenance or function, for example, by reducing the trophic support to hair cells at the nerve endings. An enrichment of rare missense variants in genes involved in axonal guidance signaling has been reported in MD [43].

In the first place, *PRKCB* gene encodes Protein kinase C (PKC), which is a serine- and threonine-specific protein kinases that can be activated by calcium and the second messenger diacylglycerol [44]. Martin-Sierra et al. [16] described that the protein encoded by *PRKCB* gene was highly expressed in the tectal cells of rat cochlea, and it showed a tonotopic expression gradient from the apical turn, where it demonstrated a strong labeling to the middle and basal turn of the cochlea. These results confirmed previous gene expression datasets generated from the mouse cochlea with higher expression in the apical and middle turns in mice [45], which matches with the low-frequency SNHL in the studied family.

*TMEM55* (transmembrane protein 55B) interacts with various proteins [46] and participates in the lysosomal dynamics [47]. Furthermore, it is part of a family of transmembrane proteins to which *TMEM132E* gene belongs, where mutations that cause DFNB99 nonsyndromic hearing loss were also described [48].

*LSAMP* (limbic-system-associated membrane protein) is a neuronal surface glycoprotein distributed in cortical and subcortical regions of the limbic system [49]; it is involved in neurite formation and outgrowth [50], and it was previously related to schizophrenia and bipolar disorder [51]. Its role in the innervation of the organ of Corti or vestibular organs has not been established.

However, *COCH*, *OTOG* and *STRC* have a higher expression in the inner ear, which suggests that changes in their protein products could cause structural modifications in the inner ear, leading to a fragile structure. *COCH* gene produces cochlin protein, which is the most abundant protein in human and mouse cochlea, and in vestibular organs of mice the second most frequent protein. Cochlin is present in the spiral ligament and spiral limbus, in the lateral wall close to the organ of Corti [52]. It is an essential structural protein to maintain the complex architecture of the lateral wall of the cochlea.

The *OTOG* gene, which encodes otogelin, is mainly expressed in acellular structures that cover the sensory inner ear epithelia: the tectorial membrane, the otoconial membranes and the cupula over the cristae ampullaris of the semicircular canals [25]. Otogelin also contributes to the horizontal top connectors and tectorial membrane-attachment crowns of OHC stereocilia, where they interact, directly or indirectly, with stereocilin [53]. The tectorial membrane is in close connection to HC stereocilia, which explains the high expression of *OTOG* in cochlear HC in the inner ear. Moreover, in the vestibular non-HC *OTOG* also had a very high expression, suggesting that these cell types could synthetize otogelin.

The *STRC* gene encodes the Stereocilin protein in cochlea and vestibule; it is associated with the gelatinous membrane overlaying the vestibular kinocilia, which suggests a role of the protein in sensing balance and spatial orientation [39]. Verpy et al. [54] reported the functional association between Stereocilin and OHC; moreover, they conclude that it is essential to the formation of horizontal top connectors, which maintain the cohesiveness of the mature OHC hair bundle, and are required for the tip-link turn over.

*DTNA* and *SEMA3D* have a relevant gene expression in neural tissues and within the inner ear. *DTNA* encodes α-dystrobrevin, which is a structural protein of the dystrophin-associated protein complex whose absence in the mouse model causes abnormalities of the blood–brain barrier and progressive edema [55]. Furthermore, Requena et al. [15] confirmed the expression of α-dystrobrevin in supporting cells by immunohistochemistry in a rat model. This also suggested that changes in this protein in the intermediate filament network can affect the motility of HC, as is reported in gene expression databases showing a high expression of *DTNA* in both cochlear and vestibular HCs.

*SEMA3D* encodes Semaphorin-3D, which belongs to the axon guidance pathway and inhibits the neural growth [56]. Gallego-Martinez et al. [43] reported an enrichment of rare missense variants in axonal guidance signaling in sporadic MD. The genes in the axon guidance pathway regulate the neurite attraction and repulsion; consequently, they patterned the auditory projections, allowing the tonotopy established by the auditory HC [57]. *SEMA3D* may be relevant to the formation or maintenance of inner ear tissues [17]; Requena et al. [58] reported that the most significant pathway in cochlear supporting cells was axonal guidance signaling, and also it was one of the most significant pathways in vestibular supporting cells, according to the differentially expressed genes in mice HC and non-HCs.

Finally, three FMD genes (*FAM136A*, *DPT* and *HMX2*) do not have a predominant gene expression in neural or inner ear tissue databases and they could not be classified in a group.

### 4.4. Limitations of Systematic Review in FMD Studies

The main weakness in FMD is that most of the SNVs and candidate genes reported were only found in single families. Additional families with pathogenic or likely pathogenic variants in the same gene segregating the phenotype would support the association between candidate genes and FMD.

A second limitation observed is that all studies that found SNVs in FMD were performed with Whole Exome Sequencing or genotyping and there are no datasets using Whole Genome Sequencing. Intronic or intergenic regions could harbor SNV, or structural variants in FMD, which are missing.

Additionally, a large set of families is necessary to perform case-control studies with a larger sample size and with patients from different families, to compare AF of the rare variants in FMD with sporadic cases as was performed in the *OTOG* study [41].

Finally, an important limitation in this systematic review was the comparison of gene expression among different databases during development and across different species. Moreover, there are few expression datasets for the vestibular organs, which did not allow us to compare between cochlear and vestibular datasets.

## 5. Conclusions

- The inheritance pattern in FMD can be AD or AR.
- Although 11 candidate genes have been reported in FMD, these genes need replication in new families and imaging studies to define which cell types are involved; they could be classified according to the gene expression profile in neural or inner ear tissues genes.

## Figures and Tables

**Table 1:** Summary of studies which describe single nucleotide variants (SNVs) selected for quantitative synthesis.

Ref.	Population	Patients	Sex	Diagnosis	Genetic Findings	
					Gene	SNV
[15]	Spanish	3	F	AAO-HNS	*FAM136A*	2:70527974G>A
					*DTNA*	18:32462094G>T
[16]	Spanish	2	M	AAO-HNS	*PRKCB*	16:23999898G>T
[38]	Korean	3	F, M	Barany Society	*COCH*	14:31349796G>A
[17]	Spanish	3	F	Barany Society	*DPT*	1:168665849G>A
		3	F, M		*SEMA3D*	7:84642128G>A
[39]	Swedish–Norwegian	3	M	SNHL and episodic vertigo	*STRC*	15:43896948G>A
[40]	Finnish	2	M	AAO-HNS	*HMX2*	10:124909634T>A
					*TMEM55B*	14:20927370G>A
[41]	Spanish	73	F, M	Barany Society	*OTOG*	11:17574758G>A
						11:17578774G>A
						11:17594747C>A
						11:17621218C>T
						11:17627548G>A
						11:17631453C>T
						11:17632921C>T
						11:17656672G>A
						11:17663747G>A
						11:17667139G>C
[42]	Iranian	2	F, M	Definite MD	*LSAMP*	3:115561402T>C *

F: female; M: male; AAO-HNS: American Academy of Otolaryngology–Head and Neck Surgery; SNHL: sensorineural hearing loss; MD: Meniere Disease; *: this SNV was not validated by Sanger sequencing.

**Table 2:** Genetic findings for each SNV (single nucleotide variant), pathogenicity and inheritance pattern.

Gene	Chr	Position	ID	cDNA	Protein	Variant Effect	Allelic Frequency ^1^		ACMG Classification	CADD Score	Inheritance Pattern
							gnomAD	Other			
*FAM136A*	2	70527974	rs690016537	c.226C>T	p.Gln76 *	Nonsense	Novel		Pathogenic (PS3, PS4, PM2, PM4, PP3)	41.00	AD
*DTNA*	18	32462094	rs533568822	c.2143G>T	p.Val715Phe	Missense	8.79 × 10^−6^	NF (CSVS)	Pathogenic (PS3, PS4, BP1)	24.90	AD
*PRKCB*	16	23999898	rs1131692056	c.275G>T	p.Gly92Val	Missense	Novel		Likely Pathogenic (PS4, PM2, PP3, PP5)	28.20	AD ^2^
*COCH*	14	31349796	-	-	-	-	Novel		Likely Pathogenic (PS4, PM2, PP2, PP3, PP5)	28.10	AD ^2^
*DPT*	1	168665849	rs748718975	c.544C>T	p.Arg182Cys	Missense	1.72 × 10^−5^	NF (CSVS)	Likely Pathogenic (PS4, PM1, PP3, PP5, BP1)	32.00	AD ^2^
*SEMA3D*	7	84642128	rs1057519374	c.1738C>T	p.Pro580Ser	Missense	Novel		Pathogenic (PS4, PM1, PM2, PP3, PP5)	25.00	AD ^2^
*STRC*	15	43896948	rs144948296	c.4027C>T	p.Gln1343 *	Nonsense	3.62 × 10^−4^	0.001 (SweGen)	Pathogenic (PSV1, PS4, PM2, PP3, PP5)	40.00	AR
*HMX2*	10	124909634	rs1274867386	c.817T>A	p.Tyr273Asn	Missense	Novel		Likely Pathogenic (PS4, PM2, PP3)	31.00	AR ^3^
*TMEM55B*	14	20927370	rs201529818	c.706C>T	p.Leu229Phe	Missense	9.56 × 10^−4^	8.2 × 10^−5^ (ExAC)	Uncertain Significance (PS4, PP3, BS1)	25.80	AR ^3^
								**CSVS**			
*OTOG*	11	17574758	rs552304627	c.421G>A	p.Val141Met	Missense	0.001288	0.004	Pathogenic (PVS1, PS4, PM2, PP3, BP1)	33.00	AR ^3^
	11	17578774	rs61978648	c.805G>A	p.Val269Ile	Missense	0.004439	0.014	Likely Benign (PS4, BP1, BP4, BP6)	19.12	AR ^3^
	11	17594747	-	-	p.Pro747Thr	Missense	Novel		Uncertain Significance (PS4, PM2, BP1, BP4)	21.90	**-**
	11	17621218	rs117005078	c.3719C>T	p.Pro1240Leu	Missense	0.005740	0.004	Likely Pathogenic (PS4, PM2, PP3, BP1)	33.00	**-**
	11	17627548	rs145689709	c.4058G>A	p.Arg1353Gln	Missense	0.004040	0.006	Uncertain Significance (PS4, PM2, BP1, BP4, BP6)	22.00	AR ^3^
	11	17631453	rs117380920	c.4642C>T	p.Leu1548Phe	Missense	0.012350	0.013	Benign (PS4, BS1, BS2, BP1, BP4, BP6)	12.42	**-**
	11	17632921	rs61736002	c.6110C>T	p.Ala2037Val	Missense	0.001207	0.004	Uncertain Significance (PS4, PM2, BP1, BP4)	7.61	AR ^3^
	11	17656672	rs76461792	c.7667G>A	p.Arg2556Gln	Missense	0.004671	0.004	Benign (PS4, BS1, BS2, BP1, BP4, BP6)	23.50	**-**
	11	17663747	rs117315845	c.8405G>A	p.Arg2802His	Missense	0.002725	0.006	Uncertain Significance (PS4, PM2, BP1, BP4, BP6)	16.79	AR ^3^
	11	17667139	rs61997203	c.8526G>C	p.Lys2842Asn	Missense	0.023350	0.019	Benign (PS4, BS1, BS2, BP1, BP6)	24.20	**-**
*LSAMP*	3	115561402	-	c.673A>G	p.Lys225Glu	Missense	Novel		Likely Pathogenic (PS4, PM2)	25.90	AR

ID: reference Single Nucleotide Polymorphism identifier; *: stop codon; NF: not found; ^1^: allelic frequencies reported in the original reports have been updated according to the available information in the last version of the reference database; gnomAD: Genome Aggregation Database; CSVS: Collaborative Spanish Variant Server; ExAC: Exome Aggregation Consortium; ACMG: American College of Medical Genetics and Genomics; CADD: Combined Annotation Dependent Depletion; AD: autosomal dominant inheritance pattern; AR: autosomal recessive inheritance pattern; ^2^: incomplete penetrance; ^3^: multiple inheritance.

## Supplementary Materials

The following are available online at https://www.mdpi.com/2073-4425/11/12/1414/s1, Table S1: Preferred Reporting Items for Systematic Reviews and Meta-Analyses (PRISMA); Table S2: Barany Society and AAO-HNS criterion to define MD.
